# Applying the quality improvement cycle to prevent cardiac surgical site infections

**DOI:** 10.1590/0034-7167-2025-0164

**Published:** 2026-03-30

**Authors:** Maria Aline Gomes de Oliveira, Ana Elza Oliveira de Mendonça, Rita de Cássia Azevedo Constantino, Jocelly de Araújo Ferreira, Kátia Regina Barros Ribeiro, Sancha Helena Lima Vale

**Affiliations:** I Universidade Federal do Rio Grande do Norte. Natal, Rio Grande do Norte, Brazil; II Universidade Federal da Paraíba. João Pessoa, Paraíba, Brazil

**Keywords:** Total Quality Management, Health Services Research, Cardiac Surgical Procedures, Surgical Site Infection, Nursing., Gestión de la Calidad Total, Investigación sobre Servicios de Salud, Procedimientos Quirúrgicos Cardíacos, Infección del Sitio Quirúrgico, Enfermería.

## Abstract

**Objectives:**

to assess the effect of a quality improvement cycle for preventing surgical site infections in adult patients undergoing cardiac surgery.

**Methods:**

a quasi-experimental before-and-after study with no control group and a quantitative approach. An improvement cycle was conducted, and data were analyzed over a one-year period at a hospital in northeastern Brazil, using nine process criteria and two outcome criteria. The analysis was performed using point estimates with a 95% Confidence Interval. Chi-square and Fisher’s exact tests were performed, with a p-value <0.05.

**Results:**

there was a significant increase in compliance with perioperative criteria. In the second assessment, a 50.8% reduction in the incidence of surgical site infections and a 74.2% reduction in mediastinitis were observed.

**Conclusions:**

the improvement cycle proved effective as a quality management tool for preventing surgical site infections.

## RESUMEN

**Objetivos:**

evaluar el efecto de un ciclo de mejora de la calidad en la prevención de infecciones del sitio quirúrgico en pacientes adultos sometidos a cirugía cardíaca.

**Métodos:**

estudio cuasiexperimental de antes y después, sin grupo control, con un enfoque cuantitativo. Se llevó a cabo un ciclo de mejora y se analizaron los datos durante un año en un hospital del noreste de Brasil, utilizando nueve criterios de proceso y dos criterios de resultado. El análisis se realizó mediante estimaciones puntuales con un Intervalo de Confianza del 95%. Se realizaron las pruebas de Chi-cuadrado y exacta de Fisher, con un valor p < 0,05.

**Resultados:**

se observó un aumento significativo en el cumplimiento de los criterios perioperatorios. En la segunda evaluación, se observó una reducción del 50,8 % en la incidencia de infecciones del sitio quirúrgico y del 74,2 % en la mediastinitis.

**Conclusiones:**

el ciclo de mejora demostró ser una herramienta eficaz de gestión de la calidad para la prevención de infecciones del sitio quirúrgico.

## INTRODUCTION

Healthcare-associated infections (HAIs) are a serious public health concern in Brazil and worldwide. These infections are acquired during the provision of care in any healthcare setting and are considered a preventable adverse event with a significant economic and social impact on the population and healthcare systems^(1)^.

According to the World Health Organization (WHO), HAIs are among the leading causes of death and increasing morbidity among hospitalized patients. According to the global report published in 2022, it is estimated that in developed countries, for every 100 patients hospitalized in Intensive Care Units, at least seven (7%) will acquire a HAI, whereas in developing countries this estimate rises to 15%^(2)^.

Surgical site infection (SSI) is directly related to surgical procedures, and its occurrence is a prevalent condition in Brazilian healthcare services^(1)^. SSI is an important complication in the postoperative period of cardiac surgery associated with several negative outcomes, including increased length of hospital stay and hospital readmission, increased morbidity and mortality rates, and is one of the most recurrent healthcare-related infections^(3,4)^.

In Rio Grande do Norte (RN), the rate of organ/cavity infection after myocardial revascularization showed increasing data from 2018 to 2022. According to the HAI report made available by the Brazilian National Health Regulatory Agency (In Portuguese, *Agência Nacional de Vigilância Sanitária* - ANVISA), the incidence density of SSI associated with cardiac surgery rose from 3.20% in 2018 to 5.80% in 2022, a percentage calculated for all notifying hospitals in RN^(5)^. In this context, the incidence density reflects the risk relationship considering length of exposure of patients undergoing the surgical procedure.

The infectious process of SSI can be identified in the first 30 days postoperatively and up to 90 days in procedures involving implants. Signs and symptoms that may be present include pain, local heat, edema, hyperemia, the presence of purulent secretion, and fever. This adverse event is classified according to the depth and tissues affected. Superficial incisional SSI involves only the skin and subcutaneous tissue. Meanwhile, deep incisional SSI affects soft tissues deep to the incision (muscle layers), and organ or cavity SSI affects any organ or cavity that was opened or manipulated during surgery, known as mediastinitis, endocarditis, pericarditis, or myocarditis in cardiac surgery^(1,6)^.

The emergence of SSIs is associated with multiple risk factors related to patients’ pre-existing conditions, such as sex, age, gender, previous infectious source, obesity, and comorbidities, but also to the surgical procedure’s environment and technical aspects. These factors include inadequate surgical prophylaxis, inadequate skin preparation (antiseptic use and shaving), poor glycemic control, poor temperature control, inadequate environment (ventilation and physical space), poor sterilization of surgical instruments and materials, length of hospital stay (preoperative hospital stay exceeding 24 hours), prolonged postoperative hospital stay, among other multifactorial causes^(3,6-8)^.

The improvement cycle aims to solve problems related to quality of care, characterized by a cyclical sequence of activities involving planning and statistical monitoring. This process allows for the redesign of care flows through the implementation of strategic changes in healthcare services, with the goal of improving patient safety and optimizing clinical outcomes^(9,10)^.

SSI prevention strategies are complex and require the integration of a series of measures to be implemented pre-, intra-, and postoperatively for safe care. Therefore, implementing best practices for SSI prevention is necessary as a strategy to reduce this event, minimize losses, and reduce health damage. This strategy offers a way to systematize care for patients undergoing cardiac surgery and establish barriers that ensure patient safety.

At the study hospital, the Healthcare-Related Infection Control Service (In Portuguese, *Serviço de Controle de Infecção Relacionado à Assistência à Saúde* - SCIRAS) has recorded the occurrence of SSIs related to cardiac surgery, especially postoperative mediastinitis, with an incidence rate of 4.41% in 2021. The lack of standardized care may be one of the contributing factors to these events.

## OBJECTIVES

To assess the effect of a quality improvement cycle for preventing SSI in adult patients undergoing cardiac surgery.

## METHODS

### Ethical aspects

The study was approved by the *Hospital Universitário Onofre Lopes* Research Ethics Committee, respecting the ethical precepts defined by Resolution 466/2012 of the Brazilian National Health Council^(11)^. The request for authorization to use and analyze secondary data is held by the healthcare service, recorded by the letter of consent, institutional authorization term for use of documents and Informed Consent Form.

### Study design, period and location

This is a quantitative study, with a quasi-experimental before-and-after design, without a control group, carried out in three periods from March 2022 to March 2023. For this, secondary data obtained from the Medical Archives and Statistics Service and SCIRAS were used at the location where the quality improvement cycle for SSI prevention and assessment of the effect of the interventions carried out was developed.

The study was conducted in the study hospital’s adult inpatient unit, surgical center, and Cardiology Intensive Care Unit (ICU), located in Natal, the capital of Rio Grande do Norte. The hospital is a private hospital complex, a reference in interventional cardiology and pediatric and adult cardiovascular surgery, and authorized by the Ministry of Health through Ordinance 234 of March 17, 2020 for heart transplantation^(12)^.

The study followed the Standards for QUality Improvement Reporting Excellence 2.0 guidelines. The use of this instrument establishes standards and structure for publishing experiences in improvement cycle projects^(13)^.

### Population and sample

From January to March 2022, preliminary data were collected to plan the improvement cycle. Subsequently, from March 2022 to March 2023, the cycle was implemented, and data for analyzing the effectiveness of actions were collected at three points during this period. Reoperations and pediatric cardiac surgical procedures were excluded. Sampling was non-probabilistic and random, consisting of the sample universe of the care team’s records in medical records, containing data on the care received in patients’ pre-, intra-, and postoperative periods as well as statistics on SSIs related to cardiac surgery in the corresponding period.

### Study protocol

The study followed the strategy of applying a cycle for planning actions to improve service quality and was developed as follows: Identification and prioritization of improvement opportunities; Analysis of the causes of identified problems; Definition of criteria for measuring the level of quality; Initial assessment of the level of quality; Planning and implementation of timely improvement interventions, the effect of which will be measured by reassessing the problem; Data reassessment to verify the effectiveness of the interventions carried out.

Prior to designing the improvement intervention plan, a healthcare quality team was established, consisting of eight members representing managers and nurses from the study site’s care areas. This team also received advisory support from SCIRAS and the institution’s Patient Safety Center. Thus, the study was conducted in three stages: 1) analysis of nonconformities before implementing the improvement cycle; 2) a prospective intervention study to be conducted through the quality improvement cycle application; 3) data reassessment from the first stage to monitor the compliance rate in cardiac surgical care before and after the improvement cycle.

### Identification and prioritization of improvement opportunities

Selection was based on an analysis of care nonconformities identified in the service. To this end, the nominal group technique was used to identify opportunities for improvement and apply the prioritization matrix using the QualiTOOL^(14)^ software prototype, in which each evaluator assigned a score of 1 to 5 points (decision criteria: minimum = 1; maximum = 5) for each improvement opportunity listed.

The prioritization matrix identified quality issues, and assessed the following criteria: whether the problem affects many patients; whether it poses a serious risk to patients; whether it can be resolved; and whether it is a viable solution. The weakness identified in the service received a maximum score across all criteria assessed by the team.

During the meeting, the high prevalence of SSI in cardiac surgery was considered by the evaluators to be the most relevant quality problem for implementing an improvement intervention.

### Analysis of causes

To analyze the care process, the various types of contributing factors were listed and grouped into four generic categories related to the patients’ profile and their risk factors, related to professionals, work processes in perioperative periods and means of monitoring infection cases.

The root cause analysis involved the interdisciplinary team’s participation that experienced the event, which provided the recovery of important information for understanding failures in different phases of the process, subsequently proposing a redesign of the assistance processes to reduce the risk of new incidents.

### Quality criteria

To construct the criteria, the Grading of Recommendations Assessment, Development and Assessment^(15)^ methodology was used, which classifies the measures to reduce SSI according to the WHO and ANVISA, applied to the reality of the service and listed in Chart 1.

**Chart 1:** Criteria for assessing the quality of cardiac surgical care, Natal, Rio Grande do Norte, Brazil, 2022

Criteria	Clarifications
C1. Bathing with a 2% chlorhexidine degerming solution and oral hygiene with 0.12% chlorhexidine in the preoperative period of cardiac surgery.	Proper hygiene and skin care before surgery reduces the risk of developing SSIs. It is recommended to perform this procedure the night before the surgical procedure and on the day of surgery.
C2. Performing trichotomy at an interval of less than or equal to two hours after the surgical incision only when necessary.	Trichotomy consists of removing hair from the area around the surgical incision. When necessary, it should be performed before surgery using an electric shaver with a single blade. Improper shaving may result in SSI.
C3. Antibiotic prophylaxis carried out up to one hour before the incision^*^.	Antibiotic administration to patients undergoing cardiac surgery as a preventive measure for SSI is considered adequate when performed one hour before the surgical incision.
C4. Surgical field antisepsis was performed with 2% chlorhexidine degerming agent followed by 0.50% alcoholic solution.	Surgical field antisepsis is the cleaning of the skin where the surgical incision will be made with antiseptic solutions, considered adequate when the skin of the surgical field is prepared with a degerming antiseptic followed by an alcoholic one.
C5. Assessment of cardiac surgical procedure time.	This involves assessing the time from the beginning to the end of the surgery. The mean time is four to five hours. The longer the surgery, the greater the risk of SSI.
C6. Performing glycemic control in the immediate postoperative period (in the first six hours after surgery).	Maintaining glycemic control is important, as hyperglycemia (increased blood glucose) is a risk factor for developing postoperative infection. This risk can be reduced by improving glycemic control (below 200 mg/dL).
C7. Use of a sterile adhesive dressing impermeable to water and air (OPSITE^®^) in the first 48 hours after surgery in patients undergoing cardiac surgery.	The use of a waterproof and airtight adhesive dressing is intended to protect the incision from exogenous contamination or inoculation of endogenous pathogens, minimizing the risk of infection and promoting a favorable healing environment. It should be noted that if the dressing exudes excessive exudate, it should be changed before the recommended time. The criterion for determining the dressing’s retention time is related to the wound’s healing process, as the inflammatory phase typically occurs two to three days post-procedure.
C8. Carrying out a preoperative checklist (checking exams, assessing skin and signs of infection upon admission).	The pre-surgical checklist is a tool used before anesthetic induction. This review, when performed properly, can prevent adverse events, including aborting the surgery. It is a recheck to ensure no critical stages or points in preoperative care are overlooked.
C9. Assessment of preoperative hospitalization time less than 24 hours.	The longer patients’ hospital stay, the greater the risk of healthcare-associated infection. Consider hospitalization adequate if < or equal to 24 hours.
C10. Incidence of SSI related to cardiac surgery.	Number of cardiac SSI diagnosed/Number of surgeries corresponding to the period X 100.
C11. Incidence of postoperative mediastinitis.	Number of postoperative mediastinitis/Number of surgeries corresponding to the period X 100.

C - Assessment criterion followed by the Arabic numerals from 1 to 11; SSI - surgical site infection; N° - number;

* Excludes antibiotic administration in infected surgeries. In this case, antibiotic use is therapeutic, not prophylactic.

This stage aimed to develop valid and reliable criteria to measure the quality of care provided to patients undergoing cardiac surgery. These criteria were subjected to content validation to ensure their adequacy.

From medical records, information was collected regarding the following data: incidence of cardiac SSI; incidence of postoperative mediastinitis obtained through statistical data from SCIRAS; and analysis of compliance with criteria for assessing the level of care quality related to cardiac surgery.

Initial assessment revealed the need to implement improvement cycle interventions. First assessment was conducted six months after initial assessment, after the cardiac SSI prevention protocol had been developed and disseminated. The second data collection was conducted two months later to assess improvement sustainability throughout data reassessment from the first stage. To assess the effect of the improvement cycle, an analysis of outcome indicators during the study period was performed on the incidence of SSI related to cardiac surgery and the incidence of postoperative mediastinitis before and after the improvement cycle.

### Interventions applied during the improvement cycle

After collection, the data were analyzed and used to plan interventions focusing on the identified quality defects. To plan interventions, improvement proposals were discussed by each member of the healthcare quality team, grouping the actions into strategic lines classified into subgroups and using the Affinity Diagram as a process management methodology, and the Gantt Chart to control the project task schedule.

The main actions defined by the team were: 1) Develop a protocol for preventing SSI in cardiac surgeries; 2) Develop a bundle for preventing SSI related to cardiac surgeries; 3) Promote educational actions for the care team for preventing cardiac SSI; 4) Implement strategies for adherence to the protocol and bundle for preventing cardiac SSI.

Understanding that care for SSI prevention goes beyond hospital environments, a discharge guidance booklet was created in the postoperative period of cardiac surgery, encouraging home self-care.

Actions to reduce variability in care and make immediate corrections were possible due to concurrent audits carried out by SCIRAS professionals, members of the improvement team and managers of care areas (inpatient unit, surgical center and ICU), allowing timely and immediate interventions through continuous monitoring of care routines.

### Analysis of results and statistics

The data were constructed in a spreadsheet to create descriptive tables and apply statistical tests. The Statistical Package for the Social Sciences temporary version 25.0 was used. Data were analyzed using absolute and relative frequency (%), using chi-square and Fisher’s exact tests (if values < 5) to compare categorical variables. A 95% Confidence Interval and a significance level (p-value < 0.05) were considered.

Data analysis was calculated from a random (representative) sample of at least 30 medical records. The results presented in a table to characterize the sample were described according to the variable, considering the level of compliance based on the measurement of criteria for assessing the quality of care in cardiac surgery.

## RESULTS

Chi-square (X^2^) and Fisher’s exact tests, with a significance level of 5%, showed a statistical association between initial assessment and first assessment of criteria related to chlorhexidine bathing, shaving, procedure time, and use of sterile dressing on the incision (C1, C2, C5, and C7), in which there was an increase in the percentage of compliance with statistical significance. In contrast, C9, which refers to hospital stay of less than 24 hours, despite an absolute improvement, did not reach the level of significance, configuring a relevant problem still present in the service even after the intervention, as shown in Table 1.

**Table 1:** Estimated quality improvement before and after intervention in the safe cardiac surgery process, Natal, Rio Grande do Norte, Brazil, 2022

Criterion	Initial assessment		1^st^ reassessment		Absolute improvement	Relative improvement	*p* value
	Conformity	+ 95%CI	Conformity	+ 95%CI			
**C1**	13.00%	12.00%	90.00%	10.70%	77.00%	85.56%	**<0.001^(1)^**
**C2**	0.00%	0.00%	20.00%	14.30%	20.00%	100.00%	**0.031^(2)^**
**C3**	73.00%	15.90%	90.00%	10.70%	17.00%	18.89%	0.095**^(1)^**
**C4**	83.00%	13.40%	90.00%	10.70%	7.00%	7.78%	0.704**^(2)^**
**C5**	67.00%	16.80%	100.00%	0.00%	33.00%	33.00%	**0.001^(1)^**
**C6**	97.00%	6.10%	100.00%	0.00%	3.00%	3.00%	1.000**^(2)^**
**C7**	67.00%	6.10%	97.00%	6.10%	30.00%	30.93%	**0.003^(1)^**
**C8**	80.00%	14.30%	97.00%	6.10%	17.00%	17.53%	0.108**^(2)^**
**C9**	27.00%	15.90%	30.00%	16.40%	3.00%	10.00%	0.774**^(1)^**

*C - Assessment criterion followed by the Arabic numerals from 1 to 11; (1) - chi-square test; (2) - Fisher’s exact test; 95%CI - 95% Confidence Interval.*

In the second reassessment after applying improvement interventions, C1, C2, C5 and C7 criteria maintained the relative improvement with statistical significance (Table 2).

**Table 2:** Quality improvement estimates before and after the intervention of the safe cardiac surgery process, Natal, Rio Grande do Norte, Brazil, 2022

Criterion	Initial assessment		1^st^ reassessment		Absolute improvement	Relative improvement	*p* value
	Conformity	+ 95%CI	Conformity	+ 95%CI			
**C1**	13.00%	12.00%	97.00%	6.10%	84.00%	86.60%	**<0.001^(1)^**
**C2**	0.00%	0.00%	40.00%	17.50%	40.00%	40.00%	**<0.001^(1)^**
**C3**	73.00%	15.90%	67.00%	16.80%	-6.00%	-8.96%	0.573^(1)^
**C4**	83.00%	13.40%	100.00%	0.00%	17.00%	17.00%	0.062^(2)^
**C5**	67.00%	16.80%	90.00%	10.70%	23.00%	25.56%	**0.028^(1)^**
**C6**	97.00%	6.10%	100.00%	0.00%	3.00%	3.00%	1.000**^(2)^**
**C7**	67.00%	6.10%	100.00%	0.00%	33.00%	33.00%	**0.001^(1)^**
**C8**	80.00%	14.30%	100.00%	0.00%	20.00%	20.00%	**0.031^(2)^**
**C9**	27.00%	15.90%	13.00%	12.00%	-14.00%	-107.69%	0.197**^(1)^**

*C - Assessment criterion followed by the Arabic numerals from 1 to 11; (1) - chi-square test; (2) - Fisher’s exact test; 95%CI - 95% Confidence Interval.*

An absolute frequency of 118 nonconformities was observed in initial assessment, and 56 in the first assessment, representing a reduction of 62 nonconformities. Although the second assessment saw an increase to 58 nonconformities, improvement is sustainable. The aggregated results of the three assessments demonstrate the progressive improvement and the consequent decrease in nonconformities.

To analyze outcome criteria C10 and C11, referring to the incidence of infection and mediastinitis, respectively, data from patients undergoing cardiac surgery provided by SCIRAS over a 12-month period were obtained to better understand the epidemiological factor.

In the first assessment, the mean SSI rate (C10) was 13.59%. The reassessment after the improvement cycle reached a 6.91% infection incidence. Therefore, comparing the two periods, the data indicate a 50.8% reduction in SSIs related to cardiac surgery, resulting in improved outcome effectiveness and improved perioperative care quality.

In relation to the occurrence of mediastinitis (C11), the mean was 5.34% in initial assessment, representing a high incidence, which decreased to 1.38% in the final assessment. Therefore, there was a reduction of approximately 74.2% compared to the initial value, falling within the spectrum reported in the literature. In June, September, November, and December 2022, there were no occurrences of postoperative mediastinitis, demonstrating a reduction in organ or cavity SSI.

## DISCUSSION

This study identified a progressive improvement in the systematization of care provided to cardiac surgical patients during the perioperative period. Measurements of the process indicators assessed in the study revealed an increase in compliance in C1 after the interventions, reaching 97% compliance with the criterion related to body bathing. Preoperative bathing with antiseptic is a stage in skin preparation that aims to reduce the microbial count on the skin and acts as an adjunct in SSI prevention.

There is no consensus in the literature regarding the effectiveness of antiseptic baths in preventing SSIs, with this strategy being reserved for major surgeries, such as cardiac surgery. Studies suggest that preoperative bathing does reduce skin contamination, but there is insufficient evidence to classify the product as the ideal choice for greater effectiveness^(16)^.

When analyzing the shaving practice, which corresponds to C2, it was found that the practice was performed inadequately, with the use of disposable blades, adapted during the study for the use of an electric shaver. After the intervention, a 40% increase in care compliance was observed compared to initial assessment, and when indicated, shaving was performed within two hours before the skin incision (operative field) with an electric shaver in the preoperative phase.

The analysis of this indicator was significantly compromised, as 60% of medical records did not record the method used and/or the time it was performed. It is worth noting that the method and the inadvertent indication for shaving compromise patient safety goals. The results of this analysis highlighted the need to strengthen good practices for professional record-keeping in patient records, ensuring the quality of information and a reliable analysis of the quality of care provided.

C4, C6, C7, and C8, in turn, achieved 100% compliance with the quality criteria. C8, related to the application of a surgical safety checklist for the preoperative period, administered by nurses in the inpatient unit before transferring patients to the operating room, is a strategy that coordinates care, identifies predictive signs of postoperative complications, reduces errors, and strengthens patient safety practices.

International research has shown that using the Safe Surgery Checklist is a low-cost, highly effective strategy in the surgical process, validating safer, more patient-centered perioperative processes and promoting improved quality of care^(17,18)^.

C4, related to surgical field antisepsis performed with 2% chlorhexidine disinfectant, followed by 0.5% alcoholic disinfectant, states that the consensus is that the effectiveness of skin preparation for the surgical procedure has a direct impact on the occurrence of SSI and depends on both the method applied and the antiseptic used.

The patients’ skin is the main source of endogenous contamination of the surgical wound. These infections are caused by a variety of pathogens, the most common in SSIs being those that comprise the skin microbiota and the area being manipulated. They implant themselves in the surgical field between the incision and its complete closure. Therefore, identifying these agents, understanding their transmission routes, and preparing the skin are essential to guide control practices, thus minimizing the risk of SSIs and improving clinical outcomes for patients^(19,20)^.

In 2009, ANVISA published nine recommended process indicators for SSI prevention^(21)^, which are regularly updated, such as preoperative hospital stay of less than 24 hours, which corresponds to the C9 measured in the study. Preoperative hospital stay is often associated with increased SSI due to colonization of the skin and mucous membranes by hospital microbiota, in addition to the risk of cross-infection through exposure to healthcare professionals.

When analyzing C9’s noncompliance, numerous factors that contributed to the negative outcome stand out. For instance, there is the demand for unscheduled surgeries resulting from hemodynamics, in which cardiac dysfunctions requiring surgical intervention are detected, referring the patient to surgical preparation and remaining in the institution for a mean of seven days. Other factors that hinder the reduction of this hospital stay are the lack of blood components necessary for surgical reserve and the unavailability of the surgical team.

To ensure reliable analysis of C3, related to prophylactic antibiotics administered up to one hour before surgical incision, the Perioperative Nursing Care Systematization form was updated to include surgical incision time. Compliance reached 90% in first assessment, with a 67% decline in the second reassessment. The most common problem was the lack of information on medication dosage. Another inadequacy was administration outside the one-hour timeframe before surgical incision.

The use of prophylactic antimicrobials in cardiac surgeries is considered one of the fundamental measures for preventing SSI, one of the main preventable adverse events and extremely harmful to patients and hospitals, as it compromises patient safety and the quality of care^(22)^. It is estimated that more than 60% of SSIs can be prevented by applying recommendations based on scientific evidence, especially with the use of surgical antibiotic prophylaxis.

Criteria related to postoperative measures, C6 and C7, achieved 100% compliance with the quality criteria. All patients in the sample performed glycemic control within the first six hours postoperatively and used a sterile, impermeable dressing within the first 48 hours postoperatively in the second reassessment after implementation of improvements. Recent review studies, published in 2022 focusing on patients undergoing surgery, indicate that strict glycemic control is ideal for reducing SSI incidence and perioperative complications^(23)^.

Surgical wound care includes maintaining a sterile dressing applied at the end of surgery and maintained for 48 hours after the surgical procedure, preventing contamination and bacterial proliferation, in addition to providing ideal conditions for healing^(24)^.

The latest updates on this topic were published by the WHO, which released global guidelines for SSI prevention in 2016^(25)^, advocating that advanced dressings should not be used in place of standard dressings in wounds healing by primary intention. Currently, there is no conclusive evidence proving the superiority of advanced dressings over standard dressings with sterile gauze.

With the implementation of prevention measures, when reassessing the SSI incidence indicator in cardiac surgery, a significant reduction was seen in relation to initial assessment, reaching 3% in September, 2.7% in October and 5% in December 2022, a percentage compatible with the mean SSI in cardiac surgery (myocardial revascularization) of 5.8% of newborns, according to the 2022 ANVISA report^(5)^.

The assessment of quality of care according to the quality framework presented by Avedis Donabedian^(26)^ is based on the assessment of three dimensions: structure; process; and outcome. These measures serve as a guide for decision-making in healthcare organizations. Other criteria used, as measurement instruments and evidence of quality improvement implemented in the study hospital, were outcome indicators related to the incidence rate of SSI and postoperative mediastinitis. Ultimately, outcome assessment is the assessment that truly captures the achievement of the proposed objective.

### Study limitations

Incomplete data on the operating room record was one of the weaknesses identified in the surgical care team’s records, hindering the analysis of care compliance and the consolidation of indicators. Also noteworthy is the difficulty in collecting retroactive data related to the quality of information in medical records and SSI diagnoses.

### Contributions to health

The results of this study indicate that the appropriate application of prevention criteria in a context of cardiac surgical intervention contributes to improving the quality of care, with a reduction in SSI rates and an improvement in the prognosis of patients undergoing the procedure.

## CONCLUSIONS

The study demonstrated the importance of applying the improvement cycle as a quality management tool, in conjunction with the science of improvement, to standardize care practices, resulting in a reduction in nonconformities in meeting surgical criteria, a reduction in infectious complication rates, and improved patient safety.

The development of improvement processes must be structured, based on scientific evidence, and aimed at reducing adverse events and strengthening the culture of safety in healthcare services. Although quality improvements have been achieved across most criteria, monitoring must be continuous to constantly improve processes, especially those that were weak and/or showed no evidence of improvement. Therefore, it is suggested that the use of quality management strategies such as improvement cycles be part of work routine and be a priority for healthcare service management.

## Data Availability

The research data are available within the article.
