# Supplementary material for: Applying the quality improvement cycle to prevent cardiac surgical site infections
Source: Rev Bras Enferm. 2026 Mar 30;79:e20260164. doi: 10.1590/0034-7167-2025-0164 (PMC13035286; doi:10.1590/0034-7167-2025-0164)
Supplement: Translation

# Translation: Aplicação do ciclo de melhoria da qualidade para prevenção de infecção de sítio cirúrgico cardíaco

**DOI:** 10.1590/0034-7167-2025-0164pt

**Authors:** Maria Aline Gomes de Oliveira, Ana Elza Oliveira de Mendonça, Rita de Cássia Azevedo Constantino, Jocelly de Araújo Ferreira, Kátia Regina Barros Ribeiro, Sancha Helena Lima Vale

**Affiliations:** I Universidade Federal do Rio Grande do Norte. Natal, Rio Grande do Norte, Brasil; II Universidade Federal da Paraíba. João Pessoa, Paraíba, Brasil

**Keywords:** Melhoria Contínua da Qualidade, Pesquisa sobre Serviços de Saúde, Procedimentos Cirúrgicos Cardíacos, Infecção de Sítio Cirúrgico, Enfermagem.

## Abstract

**Objetivos:**

avaliar o efeito de um ciclo de melhoria da qualidade para prevenção de infecção de sítio cirúrgico em pacientes adultos submetidos a cirurgia cardíaca.

**Métodos:**

estudo quase-experimental do tipo antes e depois, sem grupo controle e abordagem quantitativa. Foi realizado um ciclo de melhoria e os dados analisados dentro do período de um ano, em um hospital no Nordeste do Brasil, utilizando nove critérios de processo e dois de resultado. A análise se deu pela estimativa pontual com intervalo de confiança de 95%. Foram realizados testes quiquadrado e exato de Fisher, com p-valor <0,05.

**Resultados:**

houve aumento significativo na conformidade dos critérios perioperatórios. Na segunda avaliação verificou-se redução de 50,8% na incidência de infecção de sítio cirúrgico e 74,2% de redução de mediastinite.

**Conclusões:**

o ciclo de melhoria mostrou-se efetivo como ferramenta de gestão da qualidade para prevenção de infecções de sítio cirúrgico.

## INTRODUÇÃO

As Infecções Relacionadas à Assistência à Saúde (IRAS) constituem um grave problema de saúde pública no Brasil e no mundo. São infecções adquiridas durante a prestação de cuidados em qualquer ambiente de saúde sendo considerada um evento adverso evitável com grande impacto econômico e social para a população e sistemas de saúde^(1)^.

Segundo a Organização Mundial da Saúde (OMS), as IRAS estão entre as principais causas de morte e crescente morbidade entre os pacientes internados. De acordo com o relatório mundial publicado em 2022, estima-se que, em países desenvolvidos, a cada 100 pacientes hospitalizados em unidades de terapia intensiva, pelo menos sete (7,00%) irão adquirir uma IRAS, enquanto em países em desenvolvimento essa estimativa sobe para 15,00%^(2)^.

A Infecção de Sítio Cirúrgico (ISC) está diretamente relacionada aos procedimentos cirúrgicos, sua ocorrência é uma condição prevalente nos serviços de saúde brasileiros^(1)^. A ISC é uma importante complicação no pós-operatório de cirurgia cardíaca associado a vários desfechos negativos incluindo, o aumento no tempo de internação e readmissão hospitalar, incremento na taxa de morbimortalidade, sendo uma das infecções mais recorrentes relacionadas aos cuidados de saúde^(3,4)^.

No Rio Grande do Norte (RN), a taxa de infecção de órgão/cavidade pós revascularização do miocárdio apresentou dados crescentes nos anos 2018 a 2022. De acordo com o relatório de IRAS disponibilizado pela ANVISA, a densidade de incidência de ISC associado à cirurgia cardíaca subiu de 3,20% em 2018 para 5,80% em 2022, percentual calculado para todos os hospitais notificantes do RN^(5)^. Nesse contexto, a densidade de incidência reflete a relação do risco considerando o tempo de exposição do paciente submetido ao procedimento cirúrgico.

O processo infeccioso da ISC pode ser identificado nos primeiros 30 dias do pós-operatório e em até 90 dias em procedimentos com implantes. Os sinais e sintomas que podem estar presentes são: dor, calor local, edema, hiperemia, presença de secreção purulenta e febre. Tal evento adverso é classificado de acordo com a profundidade e tecidos acometidos. A ISC incisional superficial envolve apenas a pele e tecido subcutâneo. Enquanto a ISC incisional profunda acomete tecidos moles profundos à incisão (camadas musculares), e a ISC de órgão ou cavidade atinge qualquer órgão ou cavidade que tenha sido aberta ou manipulada durante a cirurgia, denominada mediastinite, endocardite, pericardite, ou miocardite em cirurgia cardíaca^(1,6)^.

O surgimento das ISCs está relacionado a múltiplos fatores de risco, relacionadas às condições prévias do paciente, como sexo, idade, gênero, foco infeccioso prévio, obesidade e comorbidades, mas também ao ambiente e aspectos técnicos do procedimento cirúrgico. Fatores como profilaxia cirúrgica inadequada, preparo inadequado da pele do paciente (antisséptico utilizado e tricotomia), falha de controle glicêmico, falha de controle de temperatura, ambiente inadequado (ventilação e espaço físico), falha no processo de esterilização dos instrumentais e materiais cirúrgicos, tempo de internação tempo de internação pré-operatória acima de 24 horas, tempo de internação pós-operatória prolongada entre outras causas multifatoriais^(3,6-8)^.

O ciclo de melhoria visa à resolução de problemas relacionados à qualidade do cuidado, sendo caracterizado por uma sequência cíclica de atividades que envolvem planejamento e monitoramento estatístico. Esse processo permite o redesenho dos fluxos assistenciais por meio da implementação de mudanças estratégicas nos serviços de saúde, com o objetivo de aprimorar a segurança do paciente e otimizar os desfechos clínicos^(9,10)^.

As estratégias de prevenção de ISC são complexas e requerem a integração de uma série de medidas a serem implementadas no pré, trans e pós-operatório para um cuidado seguro. Dessa forma, se faz necessário implementação de boas práticas para prevenção de ISC como estratégia para diminuir esse evento, minimizar prejuízos, reduzir danos à saúde, ao se apresentar como uma possibilidade de sistematizar a assistência aos pacientes que irão se submeter a cirurgia cardíaca, ao estabelecer barreiras que garantam a segurança do paciente.

No hospital de estudo tem sido registrado pelo Serviço de Controle de Infecção Relacionado a Assistência à Saúde (SCIRAS) ocorrência de ISC relacionada à cirurgia cardíaca, especialmente a mediastinite pós-operatória com uma taxa de incidência de 4,41% em 2021 e a ausência de padronização do cuidado pode ser um dos fatores contribuintes para esses eventos.

## OBJETIVOS

Avaliar o efeito de um ciclo de melhoria da qualidade para prevenção de infecção de sítio cirúrgico em pacientes adultos submetidos a cirurgia cardíaca.

## MÉTODOS

### Aspectos Éticos

O estudo foi aprovado pelo Comitê de Ética em Pesquisa do Hospital Universitário Onofre Lopes, em respeito aos preceitos éticos definidos pela Resolução 466/2012 do Conselho Nacional de Saúde (CNS)^(11)^. A solicitação de autorização para uso e análise de dados secundários encontra-se em poder do serviço de saúde, registrada pela carta de anuência, termo de autorização institucional para uso de documentos e Termo de Consentimento Livre e Esclarecido (TCLE).

### Desenho, período e local do estudo

Trata-se de um estudo de abordagem quantitativa, com desenho quase-experimental do tipo antes e depois, sem grupo controle, realizado em três períodos de março de 2022 a março de 2023. Para isso foram utilizados dados secundários obtidos junto ao Setor de Arquivo Médico (SAME) e ao SCIRAS do local onde foi desenvolvido o ciclo de melhoria da qualidade para prevenção de ISC e avaliação do efeito das intervenções realizadas.

A pesquisa foi realizada na unidade de internação adulto, no centro cirúrgico e na unidade de terapia intensiva cardiológica do hospital de estudo, localizado em Natal, capital do Rio Grande do Norte (RN). O Hospital é um complexo hospitalar privado, referência em cardiologia intervencionista e cirurgia cardiovascular pediátrica e adulto, habilitado pelo Ministério da Saúde por meio da portaria nº 234 de 17 de março de 2020 para transplante cardíaco^(12)^.

O estudo seguiu as diretrizes do *Standars for Quality Improvement Reporting Excellence 2.0* (SQUIRE). A utilização desse instrumento determina padrões e estrutura para a publicação de experiências em projetos de ciclo de melhoria^(13)^.

### População e Amostra

No período de janeiro a março de 2022, foram coletados dados preliminares para o planejamento do ciclo de melhoria. Em seguida, de março de 2022 a março de 2023 o ciclo foi implementado e os dados para a análise das efetividades das ações foram coletados em três momentos, dentro desse período. Foram excluídas as reoperações e os procedimentos cirúrgicos cardíacos em pediatria. A amostragem ocorreu de forma não probabilística aleatório e foi constituída pelo universo amostral dos registros da equipe assistencial nos prontuários, contendo dados do atendimento recebido no período pré, trans e pós-operatório dos pacientes, além de estatísticas de ISC relacionadas à cirurgia cardíaca no período correspondente.

### Protocolo do estudo

O estudo obedeceu a estratégia de aplicação de ciclo para planejamento de ações de melhoria da qualidade no serviço, sendo desenvolvido da seguinte forma: 1. identificação e priorização da oportunidade de melhoria; 2. análise das causas dos problemas identificados; 3. definição de critérios para medir o nível de qualidade; 4. avaliação inicial do nível de qualidade; 5. planejamento e implantação das intervenções de melhoria oportuna, cujo efeito será medido ao reavaliar o problema; 6. reavaliação dos dados para verificar a efetividade das intervenções realizadas.

Previamente ao desenho do planejamento de intervenções de melhoria, foi instituída a equipe *Quality* em Saúde formada por oito membros com representatividade de gestores e enfermeiros das áreas assistenciais do local de estudo. Essa construção também contou com o apoio consultivo da SCIRAS e NSP da instituição. Dessa forma, o estudo foi realizado em três etapas: 1) análise de não conformidades antes da implementação do ciclo de melhoria; 2) estudo prospectivo de intervenção a ser realizado por meio da aplicação do ciclo de melhoria da qualidade; 3) reavaliação dos dados da primeira etapa para monitoramento do percentual de conformidade em assistência cirúrgica cardíaca antes e depois do ciclo de melhoria.

#### 1. Identificação e priorização da oportunidade de melhoria

A escolha baseou-se em uma análise de não conformidades assistenciais identificadas no serviço. Para isso, foi utilizado a técnica de grupo nominal para identificação de oportunidades de melhoria e aplicação da matriz de priorização com uso do protótipo de software QualiTOOL^(14)^, em que cada avaliador atribuiu uma pontuação de 1 a 5 pontos (critérios de decisão: mínimo =1; máximo = 5) para cada oportunidade de melhoria elencada.

Na matriz de priorização foram identificados problemas de qualidade que foram avaliados os seguintes critérios: se o problema afeta muitos pacientes; se representa risco grave aos pacientes; se é possível solucionar e se é uma solução viável. A fragilidade encontrada no serviço atingiu nota máxima em todos os critérios avaliados pela equipe.

Durante a reunião, a alta prevalência de ISC em cirurgia cardíaca foi considerada pelos avaliadores o problema de qualidade mais relevante para a implementação de intervenção de melhoria.

#### 2. Análise das causas

Para análise do processo assistencial foram listados os diversos tipos de fatores contribuintes e agrupados em quatro categorias genéricas relacionados ao perfil do paciente e seus fatores de risco, relacionados aos profissionais, processos de trabalho nos períodos perioperatórios e ao meio de monitoramento dos casos de infecções.

A análise de causa raiz envolveu a participação da equipe interdisciplinar que vivenciou o evento, o que proporcionou o resgate de informações importantes para compreensão de falhas em diferentes fases do processo, sendo proposto posteriormente um redesenho dos processos assistenciais para reduzir riscos de novos incidentes.

#### 3. Critérios da qualidade

Para construção dos critérios utilizou-se a metodologia GRADE (*Grading of Recommendations Assessment, Development and Evaluation*)^(15)^, que classifica as medidas para redução de ISC de acordo com a OMS e ANVISA, aplicado à realidade do serviço e listados no Quadro 1.

**Quadro 1:** Critérios para avaliar a qualidade da assistência cirúrgica cardíaca, Natal, Rio Grande do Norte, Brasil, 2022

Critérios	Esclarecimentos
C1. Realização do banho com solução de clorexidina degermante a 2,00% e higiene oral com clorexidina a 0,12% no pré-operatório de cirurgia cardíaca.	A realização da higiene e cuidado adequado da pele do paciente no pré-operatório reduz o risco de desenvolvimento de ISC. É recomendado realizar na noite que antecede o procedimento cirúrgico e no dia da cirurgia.
C2. Realização de tricotomia com intervalo menor ou igual a 2 horas da incisão cirúrgica somente quando necessário.	A tricotomia consiste na remoção dos pelos da área circunscrita à incisão operatória. Quando necessário ocorrer, realizar antes do ato operatório utilizando tricotomizador elétrico com lâmina individual. A sua realização inadequada pode resultar em ISC.
C3. Profilaxia antibiótica realizada até 1 hora antes da incisão. ^*^	A administração de antibiótico nos pacientes submetidos à cirurgia cardíaca como medida preventiva para ISC. Considerar adequado quando realizado 1 hora antes da incisão operatória.
C4. Antissepsia do campo operatório realizada com clorexidina degermante a 2,00% seguida da alcoólica a 0,50%	A antissepsia do campo operatório é a limpeza com soluções antissépticas da pele onde vai ser feita a incisão cirúrgica. Considerar adequado quando for feito o preparo da pele do campo operatório com anti séptico degermante e seguido do alcoólico.
C5. Avaliação do tempo do procedimento cirúrgico cardíaco.	Trata-se da avaliação do tempo desde o início até o término da cirurgia. A média é de 4 a 5 horas. Quanto maior o tempo de operação maior o risco de ISC.
C6. Realização do controle glicêmico no pós-operatório imediato (nas primeiras 6 horas do pós-operatório).	A realização do controle glicêmico é importante, uma vez que a hiperglicemia (aumento da glicemia) é um fator de risco para desenvolvimento de infecção pós-operatória. Esse risco pode ser diminuído com a melhora do controle glicêmico, abaixo de 200mg/dL
C7. Utilização de cobertura estéril adesiva impermeável à água e ao ar (OPSITE®) nas primeiras 48 horas do pós-operatório de pacientes submetidos à cirurgia cardíaca.	A utilização do curativo adesivo impermeável à água e ao ar tem finalidade de proteger a incisão da contaminação exógena ou inoculação de patógenos endógenos de modo a minimizar o risco de infecção, além de promover um ambiente favorável para cicatrização. Ressaltando que caso o curativo apresente exsudato excessivo, deve ser trocado antes do tempo recomendado. O critério para determinação do tempo de permanência do curativo tem relação com o processo cicatricial da ferida pois a fase inflamatória geralmente ocorre de 2 a 3 dias pós procedimento.
C8. Realização *check l*ist pré-operatório (checagem de exames, avaliação de pele e sinais de infecção na admissão)	A utilização do *check list* pré-cirúrgico é uma ferramenta aplicada antes da indução anestésica. Essa revisão realizada de forma adequada pode evitar eventos adversos inclusive abortar a realização da cirurgia. É uma nova checagem para garantir que nenhuma etapa ou ponto crítico no cuidado pré-operatório seja deixado de lado.
C9. Avaliação do tempo de internação pré-operatória menor que 24h.	Quanto maior o tempo de internação do paciente maior o risco de infecção relacionada à assistência à saúde (IRAS). Considerar adequada se < ou igual 24 horas.
C10. Incidência de ISC relacionado à cirurgia cardíaca.	Nº de ISC cardíaco diagnosticados / Nº de cirurgias correspondente ao período X 100.
C11. Incidência de mediastinite pós-operatória.	Nº de mediastinites pós-operatória / Nº de cirurgias correspondente ao período X 100.

C - Critério de avaliação seguido do número arábico do 1 ao 11; ISC - Infecção de sítio cirúrgico; N° - número;

* Exclui-se a administração de antibiótico em cirurgias infectadas, nesse caso o uso do antibiótico é terapêutico e não profilático.

Esta etapa visou elaborar critérios válidos e confiáveis para medir a qualidade da assistência prestada ao paciente submetido a cirurgia cardíaca. Esses critérios foram sujeitos a validação de conteúdo para garantir sua adequação.

A partir da consulta aos prontuários, foram coletadas as informações referentes aos seguintes dados: incidência de infecção de sítio cirúrgico cardíaco, incidência de mediastinite pós-operatória obtidos através de dados estatísticos do SCIRAS e análise de conformidade dos critérios de avaliação do nível de qualidade assistencial relacionada à cirurgia cardíaca.

A avaliação inicial revelou a necessidade de implementação de intervenções do ciclo de melhoria. A primeira reavaliação foi realizada seis meses após a avaliação inicial, após elaboração e disseminação do protocolo de prevenção de ISC cardíaco. A segunda coleta foi realizada após dois meses para avaliar a sustentação da melhoria ao longo da reavaliação dos dados da primeira etapa. Para avaliação do efeito do ciclo de melhoria realizou-se uma análise de indicadores de resultado no período da pesquisa sobre a incidência de ISC relacionada à cirurgia cardíaca e incidência de mediastinite pós-operatória antes e após o ciclo de melhoria.

#### 4. Intervenções aplicadas durante o ciclo de melhoria

Após a coleta, os dados foram analisados e utilizados para o planejamento das intervenções com foco nos defeitos de qualidade identificados. Para o planejamento de intervenções foram discutidas propostas de melhoria por cada membro da equipe *Quality* em saúde e agrupado as ações em linhas estratégicas classificadas em subgrupos utilizando o diagrama de afinidades como metodologia de gestão do processo e Diagrama de Gantt para controle do cronograma das tarefas do projeto.

As principais ações definidas pela equipe foram: 1) Elaborar um protocolo para prevenção de ISC em cirurgias cardíacas; 2) Elaborar um bundle para prevenção de ISC relacionado a cirurgias cardíacas; 3) Promover ações educativas a equipe assistencial para prevenção de ISC cardíaco; 4) Implementar estratégias para adesão ao protocolo e bundle de prevenção de ISC cardíaco.

Entendendo que o cuidado para prevenção de ISC vai além do ambiente hospitalar foi elaborado uma cartilha de orientações de alta no pós-operatório de cirurgia cardíaca incentivando o autocuidado domiciliar.

As ações para redução de variabilidade do cuidado e correções imediatas foi possível devido às auditorias concorrentes realizadas por profissionais do SCIRAS, membros da equipe de melhoria e gestores das áreas assistenciais (unidade de internação, centro cirúrgico e Unidade de Terapia Intensiva- UTI) permitindo intervenções oportunas e imediatas por meio da vigilância contínua das rotinas assistenciais.

### Análise dos resultados e estatística

Os dados foram construídos em uma planilha, para realização das tabelas descritivas e aplicação de testes estatísticos. Utilizou-se o software estatístico *Statistical Package for the Social Sciences* (SPSS), versão temporária 25.0. Os dados foram analisados por meio de frequência absoluta e relativa (%), com uso dos testes Quiquadrado e exato Fisher (se valores inferiores a 5) para comparação das variáveis categóricas. Sendo considerado o intervalo de confiança (IC 95%) e o nível de significância (p-valor <0,05).

A análise dos dados foi calculada a partir da amostra aleatória (representativa) traçada de no mínimo 30 prontuários. Os resultados apresentados em tabela para caracterização da amostra foram descritos de acordo com a variável, ao considerar o nível de conformidade baseado na medição dos critérios para avaliar a qualidade assistencial de cirurgia cardíaca.

## RESULTADOS

O teste Quiquadrado (X 2) ou Exato de Fischer, atribuído um nível de significância de 5%, evidenciou associação estatística entre a avaliação inicial com a primeira reavaliação dos Critérios referente a realização do banho com clorexidina, tricotomia, tempo de procedimento e utilização de cobertura estéril na incisão (C1, C2, C5 e C7), nos quais houve aumento no percentual de conformidade com significância estatística. Em contrapartida, o C9, que diz respeito ao tempo de internação menor que 24h, apesar de ter havida melhoria absoluta, não atingiu o nível de significância, configurando um problema relevante ainda presente no serviço mesmo após intervenção, conforme apresentado na Tabela 1.

**Tabela 1:** Estimativa de melhoria da qualidade antes e depois da intervenção do processo de cirurgia segura cardiológica, Natal, Rio Grande do Norte, Brasil, 2022

Critério	Avaliação inicial		1ª Reavaliação		Melhoria Absoluta	Melhoria Relativa	Valor de *p*
	Conformidade	+ IC95%	Conformidade	+ IC95%			
**C1**	13,00%	12,00%	90,00%	10,70%	77,00%	85,56%	**<0,001^(1)^**
**C2**	0,00%	0,00%	20,00%	14,30%	20,00%	100,00%	**0,031^(2)^**
**C3**	73,00%	15,90%	90,00%	10,70%	17,00%	18,89%	0,095**^(1)^**
**C4**	83,00%	13,40%	90,00%	10,70%	7,00%	7,78%	0,704**^(2)^**
**C5**	67,00%	16,80%	100,00%	0,00%	33,00%	33,00%	**0,001^(1)^**
**C6**	97,00%	6,10%	100,00%	0,00%	3,00%	3,00%	1,000**^(2)^**
**C7**	67,00%	6,10%	97,00%	6,10%	30,00%	30,93%	**0,003^(1)^**
**C8**	80,00%	14,30%	97,00%	6,10%	17,00%	17,53%	0,108**^(2)^**
**C9**	27,00%	15,90%	30,00%	16,40%	3,00%	10,00%	0,774**^(1)^**

*C - Critério de avaliação seguido do número arábico do 1 ao 11; (1) - Teste Quiquadrado; (2) - Exato de Fisher.*

Na segunda reavaliação após aplicação de intervenções de melhorias, os critérios C1, C2, C5, C7 mantiveram a melhoria relativa com significância estatística (Tabela 2).

**Tabela 2:** Estimativas de melhoria da qualidade antes e depois da intervenção do processo de cirurgia segura cardiológica, Natal, Rio Grande do Norte, Brasil, 2022.

Critério	Avaliação inicial		1ª Reavaliação		Melhoria Absoluta	Melhoria Relativa	Valor de *p*
	Conformidade	+ IC95%	Conformidade	+ IC95%			
**C1**	13,00%	12,00%	97,00%	6,10%	84,00%	86,60%	**<0,001^(1)^**
**C2**	0,00%	0,00%	40,00%	17,50%	40,00%	40,00%	**<0,001^(1)^**
**C3**	73,00%	15,90%	67,00%	16,80%	-6,00%	-8,96%	0,573^(1)^
**C4**	83,00%	13,40%	100,00%	0,00%	17,00%	17,00%	0,062^(2)^
**C5**	67,00%	16,80%	90,00%	10,70%	23,00%	25,56%	**0,028^(1)^**
**C6**	97,00%	6,10%	100,00%	0,00%	3,00%	3,00%	1,000**^(2)^**
**C7**	67,00%	6,10%	100,00%	0,00%	33,00%	33,00%	**0,001^(1)^**
**C8**	80,00%	14,30%	100,00%	0,00%	20,00%	20,00%	**0,031^(2)^**
**C9**	27,00%	15,90%	13,00%	12,00%	-14,00%	-107,69%	0,197**^(1)^**

*C - Critério de avaliação seguido do número arábico do 1 ao 11; (1) - Teste Quiquadrado; (2) - Exato de Fisher.*

Observou-se uma frequência absoluta de 118 não conformidades na avaliação inicial e 56 na 1ª avaliação, representando uma redução de 62 não conformidades. Apesar de na 2ª avaliação ter havido um aumento para 58 não conformidades, percebe-se sustentabilidade da melhoria. Com a demonstração dos resultados, de modo agrupado, das três avaliações, ressalta-se a melhoria progressiva e a consequente diminuição das não conformidades.

Para análise dos critérios de resultado C10 e C11, referentes a incidência de infecção e mediastinite, respectivamente, foram obtidos dados de pacientes submetidos à cirurgia cardíaca fornecidos pela SCIRAS no período de 12 meses, para melhor compreensão do fator epidemiológico.

Na primeira avaliação, a taxa média de ISC (C10) correspondeu a 13,59%, já na reavaliação após ciclo de melhoria atingiu 6,91% de incidência de infecção. Logo, ao comparar os dois períodos, os dados apontam uma redução de 50,8% de ISC relacionado à cirurgia cardíaca como consequência, a eficácia do resultado e melhoria da qualidade assistencial perioperatória.

No que diz respeito à ocorrência de mediastinite (C11), na avaliação inicial, com uma média de 5,34%, configurando elevada incidência, a qual reduziu para 1,38% na avaliação final, logo houve uma redução de cerca de 74,2% em relação ao valor inicial, encontrandose dentro do espectro referido pela literatura. Nos meses de junho, setembro, novembro e dezembro de 2022, não houve nenhuma ocorrência de mediastinite pós-operatória, expressando uma redução de ISC de órgão ou cavidade.

## DISCUSSÃO

Este estudo identificou uma melhoria progressiva da sistematização do cuidado prestado ao paciente cirúrgico cardiológico nos períodos perioperatórios. As mensurações dos indicadores de processo avaliados na pesquisa revelaram um aumento de conformidades no C1 após as intervenções, atingindo 97% do cumprimento do critério relacionado ao banho corporal. O banho pré-operatório com antisséptico é uma etapa do preparo da pele que visa reduzir a contagem da microbiana da pele e atua como adjuvante na prevenção de ISC.

Não há consenso na literatura em relação à efetividade do banho antisséptico na prevenção de infecção de sítio cirúrgico (ISC), sendo tal estratégia reservada a cirurgias de grande porte, como as cirurgias cardíacas. Estudos afirmam que o banho pré-operatório de fato reduz a contaminação da pele, porém não encontraram evidências suficientes para classificar o produto como escolha ideal para maior eficiência^(16)^.

Ao analisar a prática da tricotomia, que corresponde ao C2, constatou-se que a prática era realizada de forma inadequada com uso de lâminas descartáveis, adaptada durante o estudo para uso de tricotomizador elétrico. Após a intervenção observou-se um aumento de 40% de conformidades do cuidado com relação à avaliação inicial, sendo sua realização, quando indicada, executada em até duas horas antes da incisão da pele (campo operatório) com tricotomizador elétrico na fase pré operatória.

O procedimento de análise desse indicador foi muito comprometido, fato justificado por 60% dos prontuários não possuir registro do método utilizado para este procedimento e/ou horário da sua realização. Vale ressaltar que o método e indicação inadvertida da tricotomia comprometem as metas para segurança do paciente. O resultado dessa análise, evidenciou a necessidade de fortalecer as boas práticas dos registros profissionais nos prontuários dos pacientes garantindo a qualidade das informações e a análise fidedigna da qualidade da assistência prestada.

Os critérios C4, C6, C7 e C8, por sua vez, atingiram 100% de cumprimento dos critérios de qualidade. O C8, relacionado a aplicação do *check list* de segurança cirúrgica para o período pré-operatório, aplicado na unidade de internação pelas enfermeiras antes de encaminhar o paciente para o centro cirúrgico, é uma estratégia que coordena a assistência, identifica sinais preditivos de complicações no pós-operatório, reduz erros e fortalece as práticas de segurança do paciente.

Pesquisas internacionais têm apontado que a utilização dá Lista de Verificação de Cirurgia Segura é uma estratégia custo-efetiva no processo cirúrgico, validando processos perioperatórios mais seguros e centrados no paciente promovendo melhoria da qualidade assistencial^(17,18)^.

O C4 relacionado a antissepsia do campo operatório realizado com clorexidina degermante a 2% seguida da alcoólica a 0,5%, disserta o consenso de que a eficácia da preparação cutânea para realização do ato operatório tem impacto direto na ocorrência de ISC e depende tanto do método aplicado e do antisséptico utilizado.

A pele do paciente constitui-se na principal fonte de contaminação endógena da ferida operatória. Essas infecções são causadas por uma variedade de patógenos, sendo os mais frequentes nas ISC aqueles que compõem a microbiota da pele e do sítio a ser manipulado, eles se implantam, no campo cirúrgico no período entre a incisão e o seu completo fechamento, portanto, a identificação desses agentes, a compreensão de suas vias de transmissão e preparação da pele é fundamental para orientar as práticas de controle minimizando assim o risco de ISC e melhorando os resultados clínicos dos pacientes^(19,20)^.

Em 2009, a ANVISA publicou nove indicadores de processo recomendados para prevenção de ISC^(21)^, que são atualizados regularmente, sendo eles, o tempo de internação pré-operatória menor que 24 horas, que corresponde ao C9 mensurado no estudo. A permanência pré-operatória está frequentemente associada ao aumento de ISC devido à colonização da pele e mucosas pela microbiota hospitalar, além do risco de infecção cruzada pela exposição aos profissionais de saúde.

Ao analisar a inconformidade de C9 destaca-se os inúmeros fatores que contribuíram com o resultado negativo. A exemplo, tem-se a demanda de cirurgias não programadas provenientes da hemodinâmica, onde é detectado disfunções cardiológicas que necessitam de intervenção cirúrgica e encaminham o paciente para o preparo operatório, permanecendo na instituição em média sete dias. Outros fatores que dificultam a redução desse tempo de internação, são a falta de hemocomponentes necessários para reserva cirúrgica e indisponibilidade da equipe cirúrgica.

Para análise fidedigna do C3 relacionado a antibiótico profilático até uma hora antes da incisão cirúrgica, foi atualizado a ficha de Sistematização da Assistência de Enfermagem Perioperatória (SAEP) e foi incluído o horário da incisão cirúrgica. O nível de conformidade atingiu 90% na 1ª reavaliação e apresentou um declínio para 67% na 2ª reavaliação, sendo a ausência de informação sobre dosagem do medicamento o problema mais evidenciado, outra inadequação foi a administração fora do espaço temporal de uma hora antes da incisão cirúrgica.

O uso de antimicrobianos profiláticos em cirurgias cardíacas é considerado uma das medidas fundamentais para prevenção de ISC, um dos principais eventos adversos (EA) evitáveis e extremamente prejudicial aos pacientes e hospitais, pois compromete a segurança do paciente e a qualidade da assistência^(22)^. Estima-se que mais de 60% das ISC possam ser prevenidas com a aplicação de recomendações baseadas em evidências científicas, especialmente com o uso da antibioticoprofilaxia cirúrgica

Os critérios relacionados a medidas no pós-operatório, C6 e C7 atingiram 100% de conformidade dos critérios de qualidade. Todos os pacientes da amostra realizaram controle glicêmico nas primeiras 6 horas do pós-operatório, utilizaram curativo estéril impermeável nas primeiras 48 horas do pós-operatório na 2ª reavaliação, após implementação de melhorias. Estudos de revisão recentes, publicados em 2022 com foco em pacientes submetidos a cirurgia, indicam que o controle glicêmico rigoroso é o ideal para redução da incidência de ISC e de complicações peri-operatórias^(23)^.

O cuidado da ferida operatória inclui a manutenção de curativo estéril realizado ao final da cirurgia e mantido por 48 horas após procedimento cirúrgico, evitando contaminação e proliferação bacteriana além de proporcionar condições ideais para cicatrização^(24)^.

As últimas atualizações nesse tema foram publicadas pela Organização Mundial da Saúde, que lançou em 2016 as diretrizes globais para a prevenção de ISC^(25)^, defendendo que os curativos avançados não devem ser utilizados no lugar do curativo-padrão nas feridas com cicatrização por primeira intenção. Atualmente ainda não existem evidências conclusivas que comprovem a superioridade dos curativos avançados em relação ao curativo-padrão, com gaze estéril.

Com implementação de medidas para prevenção, ao reavaliar o indicador de incidência de ISC em cirurgia cardíaca, evidenciou-se uma redução significativa em relação à avaliação inicial, chegando a 3% em setembro, 2,7% em outubro e 5% em dezembro de 2022, percentual compatível com a média de ISC em cirurgia cardíaca (revascularização do miocárdio) de 5,8% do RN, segundo relatório da ANVISA de 2022^(5)^.

A avaliação da qualidade da assistência de acordo com o referencial de qualidade apresentada por Avedis Donabedian^(26)^, apoia-se na avaliação de três dimensões estrutura, processo e resultado. Essas medidas atuam como guia para tomada de decisões das organizações de saúde. Outros critérios utilizados como instrumentos de medição e comprovação da melhoria da qualidade implementada no hospital de estudo foram os indicadores de resultado relacionados à taxa de incidência de ISC e mediastinite pós-operatória. A avaliação de resultados, é, enfim, aquela que realmente capta o cumprimento do objetivo proposto.

### Limitações do estudo

A incompletude dos dados na ficha de sala do boletim operatório foi uma das fragilidades percebida nos registros da equipe assistencial cirúrgica, o que prejudica a análise de conformidade do cuidado e consolidação dos indicadores. Destaca-se ainda, a dificuldade na coleta de dados retroativos, relacionados à qualidade das informações nos prontuários e diagnósticos médicos de ISC.

### Contribuições para área da saúde

Resultados deste estudo denotam que a aplicação adequada de critérios de prevenção em um contexto de intervenção cirúrgica cardiológica contribui para a melhoria da qualidade assistencial, com redução de índices de ISC e melhora do prognóstico de pacientes submetidos ao procedimento.

## CONCLUSÕES

O estudo comprovou a importância da aplicação do ciclo de melhoria como ferramenta de gestão da qualidade em conjunto com a ciência da melhoria para padronização de práticas assistenciais, resultando em redução de inconformidades no cumprimento dos critérios cirúrgicos, redução de taxas de complicações infecciosas e aprimoramento da segurança do paciente.

O desenvolvimento de processos de melhoria deve ocorrer de modo estruturado, baseado em evidências científicas, visando à redução de eventos adversos e ao fortalecimento da cultura de segurança nos serviços de saúde. Apesar da melhoria de a qualidade ter sido alcançada na maioria dos critérios, o monitoramento deve ser contínuo a fim de aperfeiçoar constantemente os processos, principalmente aqueles que se apresentaram frágeis e/ou não obtiveram evidências de melhoria. Assim, sugere-se que o uso de estratégias da gestão da qualidade como ciclos de melhoria faça parte da rotina de trabalho e seja uma prioridade para gestão dos serviços de saúde.

## Data Availability

Os dados da pesquisa estão disponíveis no corpo do artigo.
